# Longitudinal Circulating Nerve Growth Factor in Pancreatic Ductal Adenocarcinoma: An Exploratory Study of Postoperative Recurrence and First-Line Disease Control

**DOI:** 10.3390/biom16101433

**Published:** 2026-10-02

**Authors:** Ingrid Garajová, Graziana Digiacomo, Andrea Cavazzoni, Bing Wang, Michela Verze, Roberta Minari, Virginia Agnetti, Elisa Giovannetti

**Affiliations:** 1 Medical Oncology Unit, University Hospital of Parma, 43126 Parma, Italyvirginia.agnetti@unipr.it (V.A.); 2 Department of Medicine and Surgery, University of Parma, 43126 Parma, Italy; 3 Lab Medical Oncology, Department of Medical Oncology, Cancer Center Amsterdam, Amsterdam UMC, Vrije Universiteit, 1081 HV Amsterdam, The Netherlands; 4 Cancer Pharmacology Lab, AIRC Start-Up Unit, Fondazione Pisana per la Scienza, 56126 Pisa, Italy

**Keywords:** pancreatic ductal adenocarcinoma, nerve growth factor, NGF, liquid biopsy, recurrence, progression-free survival, CA19-9, perineural invasion, PD-L1, TGFβ

## Abstract

Nerve growth factor (NGF) signaling is involved in pancreatic ductal adenocarcinoma (PDAC) neurotropism, perineural invasion, pain, and tumor-stromal crosstalk, but its value as a circulating biomarker remains poorly defined. We retrospectively analyzed 51 patients with PDAC who underwent serial liquid-biopsy assessment across perioperative and advanced-disease timepoints. Circulating β-NGF was assessed in plasma, and samples without measurable NGF were classified as non-detectable. NGF was analyzed as both a zero-inflated continuous variable and a binary detectable/non-detectable biomarker. Phase-aligned associations with recurrence, disease-free survival, first-line progression-free survival, CA19-9, CEA, TGFβ, PD-L1, pain, and pathological perineural invasion were evaluated using non-parametric and permutation-based methods with false-discovery-rate adjustment. NGF availability was highest at first-line treatment initiation, where 9 of 40 patients had detectable NGF (22.5%). The clearest signal emerged postoperatively: patients who subsequently recurred had higher NGF values than those who did not (median 25.5 pg/mL vs. non-detectable; nominal *p* = 0.038). Detectable postoperative NGF was directionally associated with recurrence, while detectable NGF at first-line treatment initiation was associated with numerically shorter progression-free survival. Pooled active-disease measurements did not show a clear NGF increase versus postoperative samples, arguing against a generic disease-burden signal. No association remained significant after false-discovery-rate correction; therefore, all observed signals should be interpreted as exploratory and hypothesis-generating. These findings support prospective validation of circulating NGF within multimarker liquid-biopsy strategies focused on postoperative recurrence, perineural invasion-related biology, and treatment resistance in PDAC.

## 1. Introduction

Pancreatic ductal adenocarcinoma (PDAC) remains one of the most lethal solid malignancies, with a five-year relative survival of approximately 13% [1], and a disease in which anatomical staging and conventional tumor markers incompletely describe biological behavior. Two patients with similar radiological or pathological extent may experience very different postoperative courses, recurrence timing, symptom trajectories, and responses to systemic therapy. This heterogeneity reflects the fact that PDAC is not only an epithelial malignancy but also a stromal, immune, metabolic, and neural disease. Dense desmoplasia, immune exclusion, cancer-associated fibroblasts, altered extracellular matrix, peripheral nerves, and soluble mediators collectively shape tumor progression and therapeutic vulnerability [2,3,4]. In clinical practice, CA19-9 remains the most widely used circulating marker, but it is neither tumor-specific nor sufficient to capture the biological programs that drive early recurrence, perineural spread, pain, or resistance to first-line therapy. For these reasons, there is continued interest in liquid-biopsy approaches that extend beyond tumor-derived nucleic acids and include soluble proteins reflecting tumor-stromal and tumor-neural communication [5]. Within this framework, neurotrophic factors are biologically attractive because they may report processes that are clinically important in PDAC but poorly represented by routine imaging or standard serum markers [2,3,4,5,6,7,8,9,10]. Accurate diagnosis is a prerequisite for all of these considerations: distinguishing PDAC from other pancreatic malignancies, such as acinar cell carcinoma, has direct implications for prognosis, treatment strategy, and the interpretation of circulating biomarkers, because non-ductal tumors may differ in biology, marker expression, and clinical course [11].

NGF is particularly relevant in PDAC because the disease is characterized by prominent neural remodeling and perineural invasion. Early clinical and translational studies showed that activation of the NGF/TrkA axis is associated with perineural invasion and pain in pancreatic cancer, with tissue NGF expression correlating with PNI and pain, and TrkA expression linked to tumor cell proliferation, pain, and perineural spread [12,13]. Experimental work further supports a functional role for NGF in pancreatic cancer cell proliferation, invasion, tumorigenicity, and neural invasion [14,15,16,17]. More recent studies connect NGF signaling to pancreatic stellate cells, PI3K/AKT/GSK activation, metabolic reprogramming, exosomal miRNA pathways, and direct therapeutic targeting by NGF silencing, reinforcing the concept that NGF may represent a marker of tumor-microenvironment and tumor-neural crosstalk rather than a simple tumor burden measure [18,19,20]. Importantly, NGF signaling is also receptor- and context-dependent. The high-affinity TrkA receptor has generally been linked to invasive behavior and neural tropism, whereas p75NTR-related signaling may have more complex and occasionally opposing biological implications depending on ligand availability, co-receptor expression, and cellular compartment [21,22]. This receptor-level complexity is relevant for circulating biomarker interpretation because a plasma signal cannot identify the producing cell type, receptor engagement, or spatial relationship between tumor glands, nerves, fibroblasts, Schwann cells, and immune cells. The contemporary cancer-neuroscience literature now frames PDAC perineural invasion as part of a broader neural-immune-cancer niche in which tumor cells, nerves, Schwann cells, fibroblasts, myeloid cells, and soluble neurotrophic mediators interact bidirectionally [2,3,6,7,8,9,10]. Recent mechanistic work further supports this model by linking MUC15 loss to perineural invasion through IGF1R/STAT3/NGF activation, providing additional evidence that NGF-related signaling can be embedded in broader invasive programs rather than acting as an isolated pathway [23]. Much of this supporting evidence is preclinical or tissue-based rather than derived from circulating biomarker data, which is precisely the translational gap the present analysis seeks to address.

At the same time, the translation of NGF biology into clinically useful circulating biomarkers remains uncertain. PDAC liquid-biopsy research has increasingly focused on multimarker approaches, including soluble immune and stromal mediators such as TGFβ and PD-L1 [24,25], and broader liquid-biopsy strategies for pancreatic cancer [5]. However, soluble mediators differ from genomic liquid-biopsy analytes because their levels may reflect production by tumor cells, stromal cells, immune cells, endothelial compartments, nerves, or treatment-injured tissues. This is particularly relevant for NGF, which may arise from epithelial tumor cells, stellate cells, fibroblasts, inflammatory cells, or neural elements, and whose circulating concentration may change according to surgical stress, occult recurrence, tumor burden, analgesic exposure, neuropathy, or systemic inflammation. A single cross-sectional measurement may therefore obscure biologically meaningful temporal patterns. Longitudinal, phase-aligned interpretation is more appropriate: postoperative measurements may interrogate residual disease or recurrence biology, recurrence measurements may reflect re-emerging tumor-stromal activity, and first-line treatment measurements may relate to treatment resistance or progression. Despite this rationale, NGF is rarely evaluated together with longitudinal clinical timepoints, tumor markers, immune-stromal markers, and outcome measures. This creates a practical gap: NGF has strong biological plausibility in PDAC, but its measurable clinical signal in blood-based datasets remains poorly characterized. In this context, circulating NGF was selected not as a substitute for CA19-9 but as a potentially complementary soluble marker of tumor-neural and tumor-stromal biology. CA19-9 mainly reflects tumor burden and biliary/inflammatory confounding, whereas NGF may capture biological programs related to neural remodeling, stromal activation, inflammatory repair, or treatment-associated microenvironmental changes. Longitudinal assessment is therefore advantageous because the same circulating NGF value may have different implications after surgery, at radiological recurrence, or during first-line progression.

We therefore performed a phase-aligned exploratory analysis of a PDAC liquid-biopsy dataset with serial NGF measurements. The aim was not to validate NGF as a definitive prognostic test, but to determine whether detectable circulating NGF maps to clinically meaningful disease states, including postoperative recurrence, first-line progression, tumor-marker dynamics, immune-stromal biomarker variation, pain, and pathological perineural invasion. This approach was deliberately conservative. We did not assume that all NGF measurements have the same biological meaning, and we did not pool timepoints unless the analysis specifically tested whether an apparent signal could be explained by active disease in general. Instead, each comparison was aligned to the closest clinical phase in which both NGF and the comparator variable were available. This structure is important for exploratory biomarker work in PDAC because postoperative recurrence biology, advanced macroscopic disease, and progression under therapy may involve overlapping but non-identical neural, stromal, immune, and epithelial programs. By focusing on longitudinal context rather than on a single baseline value, the study was designed to generate hypotheses for future prospective liquid-biopsy studies rather than to define a ready-to-use clinical assay.

## 2. Materials and Methods

### 2.1. Study Design and Population

This retrospective exploratory cohort study included 51 patients with PDAC enrolled in a liquid-biopsy database. The source population consisted of patients with histologically or clinically confirmed PDAC who were entered into the institutional liquid-biopsy program and had at least one plasma NGF measurement with corresponding clinical annotations. Patients were included in each analysis only when a phase-aligned NGF value and the corresponding clinical, biomarker, or outcome variable were available; patients lacking the paired variable required for a specific comparison were excluded from that comparison rather than imputed. Because the cohort was assembled retrospectively from an available biomarker database, consecutive inclusion of all eligible PDAC patients cannot be guaranteed, and residual selection bias is acknowledged. Clinical, pathological, treatment, outcome, and circulating biomarker variables were extracted from the available anonymized dataset. The cohort included patients evaluated across perioperative and advanced-disease settings, allowing NGF measurements to be mapped to clinically meaningful phases rather than analyzed as a single undifferentiated biomarker pool. For operated patients, postoperative NGF measurements were interpreted in relation to subsequent recurrence and disease-free survival. For patients receiving first-line systemic therapy, NGF measurements at treatment initiation and at progression were interpreted in relation to PFS 1L (first-line progression-free survival), tumor markers, and other circulating biomarkers measured at the corresponding disease phase. This design was chosen because the biological meaning of a soluble neurotrophic factor is likely to depend on disease context: the same measured value may have different implications after macroscopic tumor removal, at radiological recurrence, or during progression under systemic treatment. No formal sample-size calculation was performed because the analysis was retrospective and hypothesis-generating. The available sample size was determined by the number of patients and biomarker measurements present in the database. Patients with insufficient clinical annotation to assign disease phase were not used for phase-specific analyses.

The overall cohort included 51 patients. Median age at diagnosis was 72.1 years (interquartile range 64.5–77.7); 23 patients were male, and 28 were female. Surgery was recorded in 27 patients, while 24 did not undergo surgery. At baseline, metastatic disease was coded in 24 patients. Pain was recorded in 20 patients. Baseline clinical and pathological characteristics are summarized in Table 1.

### 2.2. Biomarker Variables

The primary biomarker was circulating β-nerve growth factor (NGF), measured in plasma using the Human β-NGF DuoSet ELISA Development System (R&D Systems/Bio-Techne, Minneapolis, MN, USA; catalogue no. DY256-05), according to the manufacturer’s instructions and the laboratory-validated protocol. The manufacturer-reported standard-curve range for this DuoSet assay is 31.2–2000 pg/mL; however, in the local dataset, the lowest calibration standard of the laboratory protocol was 3.12 pg/mL. Samples without a measurable signal were recorded as non-detectable (zero) for categorical analyses and retained as zero-inflated values for non-parametric continuous analyses. Samples were analyzed in duplicate, and concentrations were calculated from the standard calibration curve. Measurements below the laboratory-defined lower limit of detection (LOD) or lower limit of quantification (LLOQ), depending on the laboratory procedure, were classified as non-detectable in the database. Accordingly, NGF was analyzed both as a continuous non-parametric variable and as a binary variable (detectable versus non-detectable NGF). This dual strategy was prespecified for analytical pragmatism because NGF values were sparse and zero-inflated, making assumptions of normality inappropriate and making dichotomized detectability clinically easier to interpret in small subgroups. Additional circulating biomarkers included CA19-9, CEA, TGFβ, and PD-L1, measured in plasma according to the database timepoint. Pathological variables included T stage, N stage, and perineural invasion among operated patients. Because biomarker availability differed by timepoint and compartment, each association was analyzed only among patients with paired, phase-aligned measurements for the variables being compared. To strengthen assay validity, all measurements were interpreted within the technical constraints of the plasma ELISA platform: samples were analyzed in duplicate, values were derived from the standard calibration curve, and below-threshold results were not imputed as low positive concentrations. Because NGF in biological matrices may coexist with proNGF and other neurotrophin-related species, and because commercial immunoassays may differ in antibody specificity, dynamic range, and sensitivity, the assay should be considered suitable for exploratory phase-aligned analyses but not definitive for clinical decision-making. All detectable values in the dataset were at or above the lowest calibration standard (3.12 pg/mL). The retrospective database did not retain separate run-specific LOD and LLOQ fields for each sample; therefore, samples without a measurable signal were classified as non-detectable rather than assigned an arbitrary positive value. These observations were handled as non-detectable in binary analyses and as zero-inflated values in non-parametric continuous analyses. The number of non-detectable measurements was 2/3 preoperatively, 14/20 postoperatively, 5/10 at recurrence, 31/40 at first-line treatment initiation, and 15/20 at first-line progression (Table 2). Dichotomization was used only as a complementary descriptive approach because the distribution was highly zero-inflated and the detectable fraction was small; it was not intended to replace quantitative analyses or to define a clinically validated threshold. We acknowledge that dichotomization discards quantitative information and may amplify instability in small subgroups.

### 2.3. Outcomes

Clinical outcomes included recurrence after surgery, disease-free survival (DFS), progression-free survival during first-line therapy (PFS 1L), and overall survival (OS). Because NGF measurements were not uniformly available at all timepoints, analyses were aligned to the clinical phase in which the measurement was obtained. Postoperative measurements were evaluated against subsequent recurrence and DFS when available; the exact interval between surgery, postoperative blood sampling, and documented recurrence was not uniform because sampling was embedded in retrospective clinical follow-up, so postoperative wound-healing or inflammatory effects cannot be fully excluded. Recurrence-phase measurements were evaluated against DFS and concurrent tumor markers; first-line initiation measurements were evaluated against subsequent PFS 1L and OS; and progression-phase measurements were evaluated against PFS 1L, concurrent CA19-9, CEA, TGFβ, and PD-L1. This phase-aligned strategy was intended to reduce biologically inappropriate comparisons, such as comparing a postoperative minimal-residual-disease signal with a biomarker measured during established metastatic progression. Pain and pathological PNI were treated as clinically relevant but imperfect comparators: pain is a composite clinical phenotype influenced by tumor and treatment factors, whereas pathological PNI is a spatial tissue variable that may not be captured by a soluble circulating protein measured at a different timepoint. NGF measurements were assigned to five clinical timepoints defined before analysis: preoperative; postoperative, within three months after surgery; at recurrence, when recurrence was first documented on computed tomography (CT); at the start of first-line chemotherapy, before the first cycle; and at first-line progression, when progression was first documented on CT. Because blood sampling was embedded in retrospective clinical follow-up rather than prospectively scheduled, the exact surgery-to-sampling and sampling-to-recurrence intervals varied between patients within these windows. Postoperative NGF may therefore reflect wound healing, inflammation, tissue injury, postoperative complications, or recovery, as well as disease-related processes, and this timing variability is considered a major potential source of confounding. In patients who started first-line chemotherapy for recurrent disease, the same plasma sample served as both the recurrence and the first-line baseline measurement (n = 9).

### 2.4. Statistical Analysis

The analysis was exploratory and hypothesis-generating, and reporting followed STROBE principles for observational studies. Continuous variables were summarized using medians and interquartile ranges. Phase-aligned associations between NGF and continuous variables were assessed using Spearman rank correlation with permutation-based *p*-values. Associations between NGF and binary clinical variables were assessed using Mann–Whitney tests for NGF distributions and Fisher’s exact tests for NGF detectability. For multi-level categorical variables, permutation-based Kruskal–Wallis tests were used when cell sizes allowed. Survival comparisons between patients with detectable and non-detectable NGF were explored using Kaplan–Meier estimates with 95% pointwise confidence intervals (log-log transformation) and log-rank tests; hazard ratios (HRs) were estimated with unadjusted Cox proportional-hazards models including NGF detectability as the only covariate. False-discovery-rate adjustment was applied using the Benjamini–Hochberg method. Because the analysis involved multiple phase-specific comparisons, statistical interpretation was centered on effect direction, biological coherence, and FDR-adjusted results rather than on isolated nominal *p*-values. Given the number of exploratory comparisons and the small number of NGF-positive observations at several timepoints, nominal *p*-values were interpreted only as signals requiring validation and not as confirmatory evidence. Effect sizes were reported where interpretable to avoid reliance on *p*-values alone. Because of the small cohort size, the limited number of patients with detectable NGF, and the partial overlap of patients across clinical timepoints, no multivariable models were fitted. Unadjusted hazard ratios are therefore reported with 95% confidence intervals (CIs) as descriptive estimates of uncertainty rather than as confirmatory evidence. Age- or stage-adjusted sensitivity analyses were considered but were not performed for the same reasons, as they would have produced unstable and potentially overfitted estimates. Additional sensitivity analyses compared postoperative NGF values with pooled active-disease NGF measurements to assess whether the recurrence signal reflected a generic active-disease effect or a postoperative recurrence-related pattern. The pooled active-disease category was used only as a sensitivity analysis and combined measurements obtained during recurrence, first-line treatment initiation/progression, or metastatic/advanced disease; it was not treated as a biologically homogeneous disease state. Because a patient could contribute measurements at more than one active-disease phase, and because the recurrence sample also served as the first-line baseline sample in nine patients, this pooled analysis included more than one observation per patient and was therefore interpreted descriptively only. Missing values were not imputed because missingness reflected the retrospective availability of biomarker measurements at specific clinical phases rather than random absence of isolated data points. Each table therefore reports the available denominator for the corresponding analysis, and conclusions were restricted to comparisons with clinically interpretable paired data. No nominally significant or borderline association is presented as confirmatory because all findings lost statistical significance after false-discovery-rate adjustment. 95% CIs are reported for hazard ratios (Cox model), odds ratios (exact conditional method), and Spearman coefficients (Fisher z-transformation with the Bonett-Wright standard error). For rank-biserial correlations, resampling-based intervals were unstable because of extensive ties and very small groups and are therefore not reported. The width of these intervals reflects the imprecision of the estimates, which should be refined in larger prospective cohorts with prespecified endpoints. Potential confounders included age, disease stage, tumor burden, baseline CA19-9, ECOG performance status, treatment regimen, treatment intensity, postoperative recovery, inflammation, and analgesic exposure; these variables could influence both circulating NGF and clinical outcomes but could not be incorporated into stable adjusted models in this dataset.

Regarding the handling of non-detectable values, all continuous analyses were rank-based (Spearman correlation, Mann–Whitney and Kruskal–Wallis tests). Replacing non-detectable values with any single constant below the lowest detectable concentration (for example, half of the lowest calibration standard) leaves the ranks unchanged and therefore yields identical test statistics and *p*-values; the coding of non-detectable values as zero thus does not influence the continuous analyses. Spearman correlations and Mann–Whitney comparisons involving DFS or PFS 1L treated survival times as observed values without accounting for censoring; the proportion of censored observations in these subsets was low (for example, 3 of 30 patients for PFS 1L from first-line start, and none for DFS), and these analyses were considered complementary to the log-rank and Cox analyses.

Regarding the handling of repeated observations, because sampling was retrospective, incomplete, and clinically heterogeneous, and because detectable NGF values were sparse within several phases, the dataset was not suitable for a stable mixed-effects model, time-dependent trajectory model, or patient-level slope analysis. Each clinical timepoint was therefore analyzed separately. When a patient contributed NGF measurements at more than one disease phase, each value was used only in the corresponding phase-specific comparison, and repeated measurements from the same patient were not pooled as exchangeable independent observations within a single phase-specific analysis. Residual within-patient dependence across the overall dataset cannot be fully excluded, and the study should not be interpreted as a formal model of longitudinal NGF kinetics.

## 3. Results

### 3.1. NGF Availability Across Disease Timepoints

NGF measurements were unevenly distributed across clinical timepoints. The most informative timepoints were first-line treatment initiation, post-surgical assessment, first-line progression, and recurrence. Most NGF distributions were zero-inflated, with a substantial proportion of samples recorded as non-detectable. This pattern was observed across both perioperative and advanced-disease phases and justified the parallel analysis of NGF as a continuous non-parametric biomarker and as a binary detectable/non-detectable variable. Overall, first-line treatment initiation provided the largest analyzable subgroup, whereas preoperative and late progression/current-status measurements were sparse and were therefore considered descriptive only. The availability pattern also determined which clinical questions could be tested with interpretable denominators: postoperative measurements were most suitable for recurrence-related analyses, first-line start measurements for subsequent PFS 1L, and progression measurements for concurrent tumor-marker associations. Table 2 summarizes the distribution of circulating NGF across the available disease timepoints.

### 3.2. Phase-Aligned Associations Between NGF and Clinical or Biomarker Variables

The strongest phase-aligned signal was observed in the post-surgical setting. NGF after surgery differed according to subsequent recurrence status: patients who developed recurrence had higher NGF values than those without recurrence (median 25.5 vs. 0.0 pg/mL; rank-biserial r = 0.47; nominal *p* = 0.038). This finding did not remain significant after false-discovery adjustment (*q* = 0.55), but it was the most clinically coherent association in the dataset. In sensitivity analyses designed to maximize sample size, broader pooling of all active-disease NGF measurements did not demonstrate a clear increase compared with post-surgical NGF values. Thus, the signal appeared more consistent with a postoperative recurrence-related pattern than with a generic marker of macroscopic active disease. Because postoperative sampling intervals were not standardized, this observation should be viewed as a recurrence-associated signal requiring prospective confirmation with predefined sampling windows, not as proof of minimal residual disease or a validated recurrence-prediction threshold. The complete phase-aligned association screen is provided in Supplementary Table S1, and the postoperative versus active-disease sensitivity analyses are detailed in Supplementary Table S2.

At first-line progression, NGF showed a moderate positive correlation with CA19-9 measured at progression (Spearman rho = 0.45; *p* = 0.089). NGF at first-line progression also correlated inversely with PFS 1L (rho = −0.39; *p* = 0.105), and NGF at first-line treatment initiation showed a similar inverse association with PFS 1L (rho = −0.31; *p* = 0.107). NGF at recurrence showed an inverse association with DFS (rho = −0.57), but this analysis included only nine patients and remained statistically unstable.

No convincing phase-aligned association was observed between NGF and pain, TGFβ, PD-L1, or pathological perineural invasion. Although all NGF-positive post-surgical patients with available pathological data had perineural invasion, perineural invasion was also frequent among NGF-zero patients; therefore, the association was not statistically robust (Table 3). These negative findings should be interpreted in the context of limited paired sample size, high prevalence of PNI among operated cases, assay detectability constraints, and the biological separation between a circulating soluble protein and spatial tissue phenotypes.

### 3.3. NGF Positivity and Clinically Relevant Variables

When NGF was dichotomized as positive versus zero, several associations were directionally consistent with a more aggressive disease phenotype, but none survived false-discovery adjustment. Post-surgical NGF positivity was associated with a lower proportion of patients without recurrence (1/6 among NGF-positive vs. 9/13 among NGF-zero; *p* = 0.057). NGF positivity at first-line treatment initiation was associated with numerically shorter PFS 1L (median 3.1 vs. 6.1 months; *p* = 0.069). At first-line progression, NGF-positive patients had markedly higher CA19-9 values at progression (median 13,666 vs. 236; *p* = 0.089) as reported in Table 4.

### 3.4. Survival Analyses

**Figure 1:**
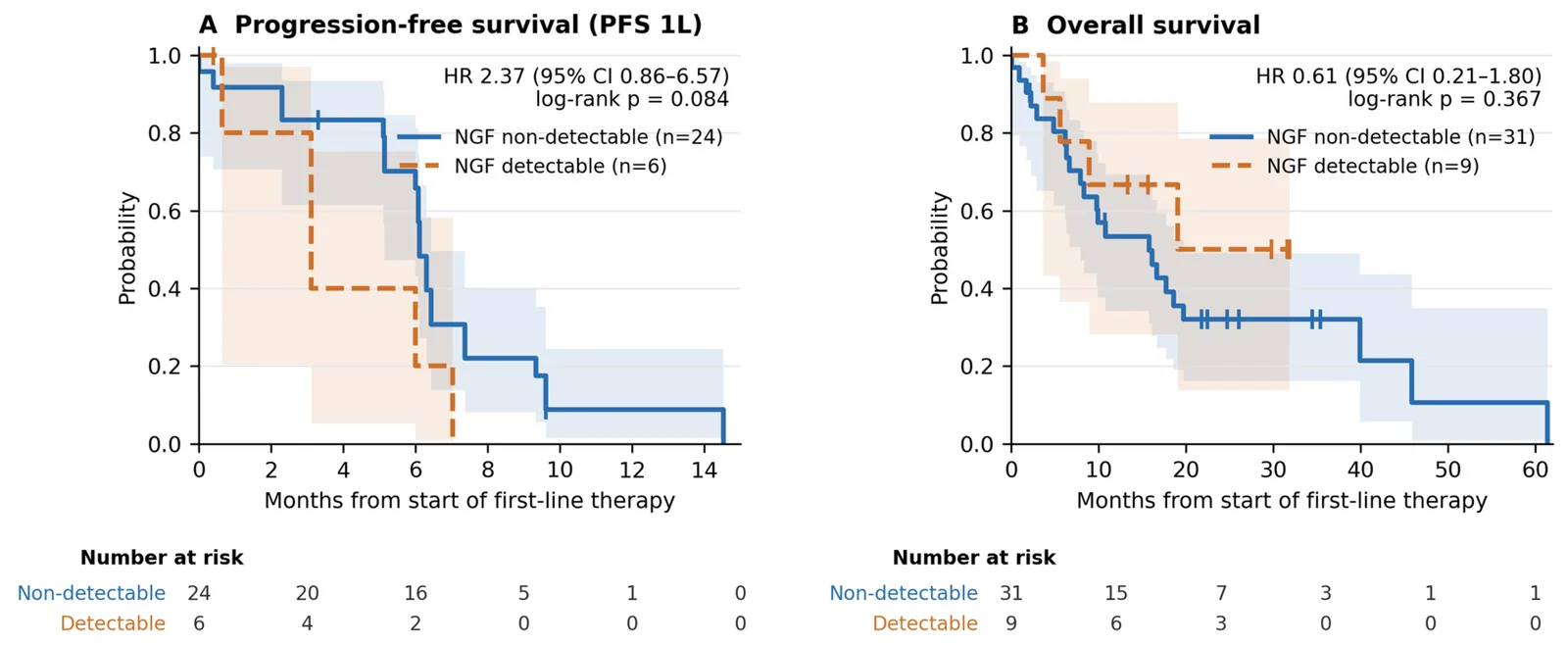
Kaplan–Meier estimates according to circulating NGF detectability at the start of first-line chemotherapy. (**A**) First-line progression-free survival (PFS 1L; n = 30, 27 events). (**B**) Overall survival (n = 40, 27 events). Shaded areas indicate 95% pointwise confidence intervals (log-log transformation); tick marks indicate censored observations. Hazard ratios (HR) are from unadjusted Cox proportional-hazards models and *p* values from the log-rank test. Numbers at risk are shown below each panel. These analyses are exploratory and were not adjusted for potential confounders.

Survival analyses were limited by the small number of NGF-positive patients and by outcome availability at each timepoint. The most relevant signal was observed for NGF at first-line treatment initiation and PFS 1L. Among 30 evaluable patients, NGF positivity was associated with a numerically higher risk of progression, with an unadjusted Cox HR of 2.37 (95% CI 0.86–6.57; log-rank *p* = 0.084; Figure 1A). NGF positivity was not associated with OS in the first-line initiation cohort (HR 0.61, 95% CI 0.21–1.80; log-rank *p* = 0.367; Figure 1B), nor was NGF after surgery associated with OS, although only two deaths were available in that subset (Table 5). These hazard ratios are unadjusted descriptive estimates and should not be interpreted as precise independent prognostic effects. The width of the confidence intervals underlines this imprecision.

## 4. Discussion

This exploratory analysis suggests that circulating NGF may capture selected, timepoint-dependent aspects of PDAC biology, particularly postoperative recurrence risk and shorter first-line disease control. The findings are not statistically definitive after correction for multiple testing, but they are clinically coherent and biologically plausible. The central observation is not that circulating NGF is globally higher in all active disease states. Instead, the signal appears strongest when NGF is interpreted in relation to clinical phase, especially after surgery and around first-line disease control. This distinction matters because soluble mediators in PDAC may not behave like simple tumor-burden markers. They may reflect transient or compartment-specific processes such as residual microscopic disease, wound-healing and stromal remodeling, early neural reactivation, inflammatory recruitment, or treatment-induced changes in the tumor microenvironment. The present data therefore support a cautious model in which circulating NGF is best viewed as a candidate component of a longitudinal multimarker strategy rather than as a standalone diagnostic or prognostic test. Accordingly, all references to residual disease, treatment resistance, and neural remodeling should be interpreted as biological hypotheses generated by phase-aligned patterns and the prior mechanistic literature, not as mechanisms demonstrated by the present circulating biomarker dataset.

Accordingly, the present work should be interpreted strictly as an exploratory and hypothesis-generating analysis. The nominal associations reported here are intended to prioritize biological hypotheses and future validation strategies, not to establish a clinically actionable NGF threshold, prove causality, or support immediate biomarker-guided treatment decisions.

The post-surgical association between NGF and recurrence is the most relevant clinical observation. Patients who recurred had higher post-surgical NGF values, and NGF positivity after surgery was directionally associated with recurrence. Importantly, this pattern was not reproduced by simply pooling all active-disease timepoints, which argues against an overly broad interpretation of NGF as a generic disease-burden marker. Rather, measurable NGF after surgery may reflect residual tumor-microenvironment activity or early recurrence biology in a subset of patients. However, the present data cannot distinguish residual disease from postoperative inflammation, tissue repair, or other host-response mechanisms. This hypothesis is consistent with the established relationship between NGF/TrkA signaling, perineural invasion, pain, and invasive growth in PDAC [12,13,14,15]. It is also consistent with evidence that neural remodeling can occur early during pancreatic carcinogenesis and may interact with adrenergic and neurotrophin-dependent programs that support tumor progression [7,26,27]. From a clinical standpoint, this raises the possibility that postoperative NGF could identify a biological state not fully captured by conventional postoperative variables, although the current sample size is far too small to support clinical use. Prospective studies should therefore evaluate NGF together with CA19-9 kinetics, circulating tumor DNA, inflammatory mediators, immune-stromal proteins, and standardized pathological PNI scoring.

The signal linking NGF with shorter disease control during first-line therapy also deserves attention. NGF positivity at first-line initiation was associated with numerically shorter PFS, and NGF at progression correlated positively with CA19-9. These findings position NGF detectability within a progression or treatment-resistance phenotype rather than as a simple baseline prognostic marker. Experimental studies support this interpretation by showing that NGF signaling can promote invasion, neural tropism, tumor growth, stellate-cell crosstalk, and metabolic adaptation [16,17,18,19]. Consistently, direct preclinical targeting of NGF with gold nanocluster-assisted NGF siRNA suppressed pancreatic tumor growth in subcutaneous, orthotopic, and patient-derived xenograft models, consistent with a role for NGF as a functional mediator rather than only a correlative marker [20]. The newly reported MUC15-IGF1R/STAT3/NGF pathway further strengthens the rationale for viewing NGF as part of an invasion-associated signaling network in PDAC [23]. Although that study is tissue- and mechanism-oriented, it supports the broader premise that NGF-linked programs may become clinically relevant when aligned to biologically meaningful disease states. In other tumor models, fibroblast-derived NGF has also been implicated in reciprocal tumor-stroma signaling and aggressive behavior, reinforcing the principle that NGF may be a stromal as well as tumor-derived signal [28]. These observations do not prove that circulating NGF is causally related to first-line resistance in PDAC, but they provide a biologically plausible context for prospective testing. Thus, treatment resistance is proposed here as a biological hypothesis generated by the timing and direction of the signal, not as a demonstrated mechanism.

The absence of a robust association between circulating NGF and pathological perineural invasion should be interpreted cautiously. Pathological perineural invasion was available only in the operated subset and was common even among NGF-zero patients, limiting discriminatory power. Moreover, circulating NGF cannot resolve the spatial organization of tumor-neural interactions or the relative contribution of TrkA- and p75NGFR-mediated signaling within the pancreatic tumor microenvironment. A negative or weak blood-based association therefore does not contradict prior pathological and mechanistic evidence linking NGF-related pathways to neural invasion; rather, it highlights the challenge of translating a spatial, receptor-dependent process into a circulating biomarker [2,3,6,7,8,9,10,21,22]. PNI is increasingly understood as a dynamic ecological process involving cancer cells, axons, Schwann cells, immune cells, stromal cells, extracellular matrix, and axon-guidance or neurotrophic cues [2,3,6,7,8,9,10,29,30,31,32]. A plasma NGF measurement may capture only one soluble output of this process and may be temporally dissociated from histological PNI recorded at surgery.

At the molecular level, the NGF/PNI axis in PDAC is best understood as a reciprocal tumor-nerve-stroma program. Tumor cells and stromal cells, including pancreatic stellate cells and fibroblast-rich compartments, can produce NGF, which may activate TrkA-dependent downstream signaling in cancer cells and neural elements. These signals converge on pathways such as MAPK/ERK, PI3K/AKT/GSK, and STAT3, promoting cell survival, migration, invasion, metabolic adaptation, neurite outgrowth, and the attraction or remodeling of peripheral nerve fibers [7,14,15,16,17,18,19,20,23]. In parallel, Schwann cells, axon-guidance cues, inflammatory mediators, and extracellular-matrix remodeling can create a permissive neural niche that facilitates cancer-cell tracking along nerves [2,3,6,7,8,9,10,26,27,29,30,31,32]. The p75NGFR/p75NTR receptor adds further complexity because NGF signaling may differ according to receptor balance, cell type, and microenvironmental context [21,22]. This mechanistic framework helps explain why circulating NGF may show postoperative or progression-related signals while still correlating imperfectly with histological PNI, which is a spatial tissue phenotype rather than a circulating analyte.

This interpretation is consistent with recent clinical and translational syntheses of PDAC, which frame treatment outcome, tumor microenvironment, neural remodeling, and perineural invasion as interconnected rather than isolated domains [2,3,4,6,7,8,9,10,23,29,30,33]. Contemporary reviews on perineural invasion emphasize that tumor-neural interactions involve multiple molecular pathways and may represent therapeutic vulnerabilities, while broader neural-immune-cancer reviews place neurotrophic factors within reciprocal circuits linking nerve remodeling, immune suppression, stromal activation, and therapeutic resistance [10,29,30,33]. These data reinforce the need to interpret circulating NGF in relation to receptor context and the broader neuro-stromal and neuro-immune microenvironment, while recognizing that liquid-biopsy measurements remain indirect readouts of this biology. Recent cancer-neuroscience reviews have also proposed neural invasion-related biomarkers, including circulating extracellular vesicles, plasma neuroactive factors, and inflammatory mediators, as potential tools for dynamic monitoring of the tumor-nerve-immune axis [10,29,30,33]. The present study fits within this translational direction but remains an early exploratory step. Its main contribution is to show that serial NGF measurement is feasible in a clinical PDAC liquid-biopsy dataset and that the most coherent signals emerge when measurements are aligned to postoperative recurrence and first-line disease-control states.

Similarly, the lack of consistent associations with pain, TGFβ, and PD-L1 does not exclude biological interaction. Pain is multifactorial in PDAC and can be affected by tumor location, biliary obstruction, analgesic use, neural invasion, treatment status, chemotherapy-induced neuropathy, and systemic inflammatory factors. The contemporary cancer pain literature increasingly recognizes that pain intensity and treatment need are shaped by peripheral nerve remodeling, nociceptive sensitization, inflammation, and psychosocial factors, making a single circulating biomarker unlikely to capture the full phenotype [34]. TGFβ and PD-L1 reflect overlapping but distinct stromal and immune axes in prior liquid-biopsy work in PDAC [24,25]. The present dataset was not powered to define biomarker networks or to test interactions between NGF, immune checkpoints, stromal mediators, and pain. Its value is therefore exploratory: it identifies where NGF fits most plausibly into future multimarker models. Future studies should collect serial NGF, CA19-9, inflammatory markers, immune-stromal markers, treatment exposure, analgesic use, neuropathy status, and standardized pain scores at predefined timepoints. The same caution applies to pathological PNI: the absence of a robust association may reflect limited paired sample size, high PNI prevalence among operated cases, assay detectability constraints, temporal mismatch between tissue and plasma measurements, or the biological complexity of translating a spatial tissue phenotype into a soluble circulating readout.

The main limitation is sample size and no external validation cohort. Although the overall database included 51 patients, NGF availability varied substantially by timepoint, and NGF positivity was uncommon. This produced small analytical subsets, wide uncertainty, and limited power after false-discovery-rate adjustment. The retrospective design also introduces unavoidable heterogeneity in sampling times, treatment status, clinical indication for biomarker assessment, and follow-up completeness. Another limitation is that circulating NGF was measured as a soluble biomarker without paired spatial profiling of tumor tissue, nerve density, Schwann-cell activation, TrkA/p75NTR expression, or immune-cell localization. Therefore, the analysis cannot identify the cellular source of circulating NGF or determine whether plasma NGF reflects tumor-derived secretion, stromal production, neural remodeling, systemic inflammation, or treatment-related tissue injury. Finally, the study should not be interpreted as a validation of a clinical cut-off. The binary detectable/non-detectable analysis was used to handle the zero-inflated distribution and to generate clinically readable exploratory comparisons. These limitations are substantial, but they are consistent with the study’s purpose: to generate hypotheses and define the most promising clinical phase for future NGF testing. In addition, the absence of guaranteed consecutive enrollment, the lack of standardized postoperative sampling intervals, and the inability to adjust for age, stage, tumor burden, ECOG performance status, treatment regimen, CA19-9, analgesic exposure, or inflammatory status mean that residual confounding cannot be excluded. The small number of NGF-detectable cases also limits the interpretability of confidence intervals and precludes robust adjusted modeling. Assay-related limitations should also be acknowledged. Published work has reported serum NGF measurement by ELISA in pancreatic cancer [35], but technical studies have emphasized that NGF/proNGF interference can affect immunoassay interpretation and that alternative platforms, including NGF/proNGF-selective immunoassays, immunoaffinity capillary electrophoresis, and immunoaffinity LC-MS/MS, may improve analytical specificity or sensitivity in future validation studies [36,37,38]. The limited number of patients at some individual timepoints reduces statistical power, increases the uncertainty of subgroup comparisons, and makes effect estimates sensitive to single observations. Therefore, subgroup findings should be read as prioritization signals for future studies rather than reliable estimates of association. Selection and immortal-time bias should also be considered: patients had to survive, remain under follow-up, and have plasma available to contribute samples at later timepoints such as recurrence, first-line initiation, or first-line progression, and these patients may differ systematically from those without such samples. This may distort associations with survival endpoints. In addition, first-line PFS and progression-phase analyses may be confounded by chemotherapy regimen, dose intensity, treatment interruptions, metastatic burden, performance status, and subsequent therapies, all of which could influence both circulating NGF and disease control; the cohort was not powered for formal adjustment for these factors.

Despite these limitations, the study has practical value. It evaluates circulating NGF longitudinally across clinically meaningful timepoints rather than as an isolated measurement, and it analyzes NGF in parallel with CA19-9, CEA, TGFβ, PD-L1, pain, pathological perineural invasion, recurrence, DFS, PFS, and OS. The analysis also avoids overfitting by not forcing multivariable models into a sparse dataset. The findings can inform the design of prospective translational studies in three ways. First, postoperative sampling should be prioritized because the strongest signal was recurrence-related rather than simply active-disease-related. Second, NGF should be tested as part of multimarker panels rather than alone, ideally integrating tumor burden markers, immune-stromal proteins, ctDNA, extracellular vesicles, and digital pathology measures of PNI. Third, mechanistic companion studies should distinguish circulating NGF from local NGF/TrkA and p75NTR signaling in tissue, because receptor balance and cellular source may determine whether NGF-associated biology reflects invasion, neural repair, immune modulation, or treatment-induced remodeling. These design principles would allow future studies to determine whether circulating NGF is merely a marker of selected disease states or a clinically actionable readout of tumor-neural crosstalk. This is particularly relevant for studies embedded in routine oncology workflows, where biomarker timing must be clinically feasible and analytically interpretable.

More broadly, the present work illustrates the practical challenges of translating biologically complex neurotrophic signaling into clinically usable circulating biomarkers. In pancreatic cancer, circulating proteins are unlikely to reflect a single cellular source or a single pathological process. Instead, they may summarize overlapping contributions from tumor cells, stromal compartments, immune cells, neural structures, treatment effects, and systemic host responses. For this reason, the interpretation of NGF should remain contextual and longitudinal. A detectable value may have different implications after surgery, during recurrence, or at progression under systemic therapy, and future studies should be designed to preserve this clinical context rather than collapse all samples into a single biomarker category.

From a translational perspective, these considerations support a stepwise validation strategy. First, exploratory datasets such as the present one can identify the timepoints at which a biomarker appears most informative. Second, prospective cohorts can test whether the same pattern is reproducible using predefined sampling windows and standardized clinical annotations. Finally, integrated analyses combining soluble biomarkers, tumor markers, imaging, pathology, and molecular liquid-biopsy platforms may clarify whether NGF adds independent information or mainly reflects broader tumor-microenvironment activity. In the longer term, molecular and gene-based imaging strategies, such as magnetic resonance reporter systems based on magnetosome-associated proteins, may provide complementary non-invasive readouts of cellular and molecular processes [39]; however, these approaches remain experimental and do not provide direct evidence regarding circulating NGF. This conservative approach is particularly appropriate for small retrospective biomarker studies, where biological plausibility is strong but clinical implementation requires external validation.

## 5. Conclusions

In this exploratory PDAC liquid-biopsy cohort, circulating NGF showed clinically coherent but non-definitive, clinical context-dependent associations with recurrence after surgery, shorter first-line disease control, and higher CA19-9 at progression. The results, none of which remained significant after false-discovery-rate correction, support the hypothesis that circulating NGF may be most informative when interpreted longitudinally and in relation to disease phase, rather than as a generic marker of tumor burden. The absence of robust associations with pain, PD-L1, TGFβ, or pathological PNI should not be interpreted as evidence against NGF-related neural biology in PDAC but rather as a reminder that circulating biomarkers are indirect and compartmentally limited readouts of complex tumor-neural interactions. Future prospective studies should validate NGF in predefined perioperative and advanced-disease cohorts, incorporate standardized sampling windows, and combine soluble NGF with CA19-9, ctDNA, extracellular vesicles, immune-stromal proteins, digital pathology, and detailed PNI assessment. Such studies will be required to determine whether NGF can contribute to clinically useful recurrence-risk stratification or treatment-resistance monitoring in PDAC. Until such validation is available, these results should be regarded as hypothesis-generating evidence supporting biologically informed sampling strategies rather than immediate clinical decision-making. Future studies should also include assay-level validation, ideally comparing the current plasma ELISA approach with more sensitive or more selective NGF platforms and reporting assay precision, recovery, lower detection/quantification limits, and the frequency of values below those limits.

## Figures and Tables

**Table 1:** Baseline characteristics of the study cohort.

Characteristic	Category	Value
Age at diagnosis, years	Median (IQR)	72.1 (64.5–77.7)
Sex	Male	23/51 (45.1%)
	Female	28/51 (54.9%)
Pain	Yes	20/51 (39.2%)
	No	31/51 (60.8%)
Tumor site	Head	34/51 (66.7%)
	Body	8/51 (15.7%)
	Tail	9/51 (17.6%)
Disease status at baseline	Metastatic	24/51 (47.1%)
Surgery	Yes	27/51 (52.9%)
	No	24/51 (47.1%)
Adjuvant therapy	Yes	20/25 (80.0%)
	No	5/25 (20.0%)
Pathological T stage	T0	1/27 (3.7%)
	T1	3/27 (11.1%)
	T2	11/27 (40.7%)
	T3	11/27 (40.7%)
	T4	1/27 (3.7%)
Pathological N stage	N0	13/27 (48.1%)
	N1	9/27 (33.3%)
	N2	5/27 (18.5%)
Perineural invasion	Yes	20/25 (80.0%)
	No	5/25 (20.0%)
Postoperative recurrence	Yes	15/27 (55.6%)
	No	12/27 (44.4%)
Death during follow-up	Yes	30/51 (58.8%)
	Alive	21/51 (41.2%)

Note: Values are N (%) unless otherwise stated. Percentages use the available denominator shown in each cell. IQR, interquartile range; NGF, nerve growth factor; PNI, perineural invasion.

**Table 2:** Availability and distribution of circulating NGF by timepoint.

Timepoint	N Measured	Detectable NGF	Median (IQR)	Range
Preoperative	3	1/3	0.0 (0.0–26.2)	0.0–52.5
Postoperative	20	6/20	0.0 (0.0–31.9)	0.0–1217.2
At recurrence	10	5/10	81.6 (0.0–283.5)	0.0–1318.5
First-line start	40	9/40	0.0 (0.0–0.0)	0.0–1773.0
First-line progression	20	5/20	0.0 (0.0–5.0)	0.0–1277.7

**Table 3:** Main phase-aligned associations involving circulating NGF.

Timepoint	NGF Measure	Outcome/Variable	Test	N	Effect (95% CI)	*p*	*q*
Postoperative	Postoperative NGF	Recurrence	Mann–Whitney	19	0.47	0.038	0.55
First-line start	NGF at first-line start	Disease status	Permutation Kruskal–Wallis	40		0.071	0.55
First-line progression	NGF at progression	CA19-9 at progression	Permutation Spearman	15	0.45 (−0.10 to 0.79)	0.089	0.55
First-line progression	NGF at progression	PFS 1L	Permutation Spearman	18	−0.39 (−0.73 to 0.11)	0.105	0.55
First-line start	NGF at first-line start	PFS 1L	Permutation Spearman	30	−0.31 (−0.61 to 0.07)	0.107	0.55
Recurrence	NGF at recurrence	DFS	Permutation Spearman	9	−0.57 (−0.91 to 0.21)	0.107	0.55
Postoperative	Postoperative NGF	Pathological PNI	Mann–Whitney	20	0.38	0.162	0.55

Note: In Table 3, the Effect size column reports rank-biserial r for Mann–Whitney tests (positive values indicate higher NGF in patients with recurrence or PNI) and Spearman rho with 95% CI (Fisher z-transformation, Bonett-Wright standard error) for Spearman correlation tests. For the permutation Kruskal–Wallis test, no single effect-size estimate is shown. *q* values are Benjamini–Hochberg false-discovery-rate-adjusted *p* values.

**Table 4:** Focused comparisons by circulating NGF detectability.

NGF Measure	Clinical Variable	N	Summary	Effect (95% CI)	*p*	*q*
Postoperative NGF	Recurrence	19	No recurrence: 1/6 NGF-positive vs. 9/13 NGF-zero	0.10 (0.002–1.33)	0.057	0.52
NGF at first-line start	PFS 1L	30	Median PFS: 3.1 months if NGF-positive vs. 6.1 months if NGF-zero	0.49	0.069	0.52
NGF at progression	CA19-9 at progression	15	Median CA19-9: 13,666 if NGF-positive vs. 236 if NGF-zero	−0.59	0.089	0.52
NGF at first-line start	DFS	13	Median DFS: 6.1 months if NGF-positive vs. 14.4 months if NGF-zero	0.55	0.099	0.52

Note: In Table 4, the Effect size column reports the conditional maximum-likelihood odds ratio with exact 95% CI (odds of remaining recurrence-free, NGF-positive vs. NGF-zero) for binary clinical variables and rank-biserial r for continuous variables compared between NGF-positive and NGF-zero groups. Positive rank-biserial values indicate lower values in the NGF-positive group. *q* values are Benjamini–Hochberg false-discovery-rate-adjusted *p* values.

**Table 5:** Exploratory survival analyses by circulating NGF detectability.

NGF Measure	Endpoint	N	Events	HR (95% CI)	Log-Rank *p*
NGF at first-line start	PFS 1L	30	27	2.37 (0.86–6.57)	0.084
NGF at first-line start	DFS	13	13	2.22 (0.71–7.00)	0.168
NGF at first-line start	OS	40	27	0.61 (0.21–1.80)	0.367
NGF at first-line progression	PFS 1L	18	17	1.50 (0.47–4.72)	0.430
Postoperative NGF	OS	20	2	2.24 (0.14–35.90)	0.560
NGF at first-line progression	OS	18	16	1.40 (0.43–4.61)	0.577
Postoperative NGF	DFS	10	10	1.32 (0.38–4.63)	0.620

Note: HR, unadjusted hazard ratio for NGF-detectable versus NGF-non-detectable patients from Cox proportional-hazards regression; *p* values are from the log-rank test. DFS was available only for patients who recurred, so all DFS observations are events.

## Data Availability

The data are not publicly available due to privacy and ethical restrictions involving clinical patient-level data. De-identified data may be available from the corresponding author upon reasonable request and subject to institutional and ethical approvals.

## Supplementary Materials

The following supporting information can be downloaded at https://www.mdpi.com/article/10.3390/biom16101433/s1. Table S1, phase-aligned circulating NGF association screen; Table S2, sensitivity analyses comparing postoperative and active-disease circulating NGF measurements.
