# Supplementary material for: Longitudinal Circulating Nerve Growth Factor in Pancreatic Ductal Adenocarcinoma: An Exploratory Study of Postoperative Recurrence and First-Line Disease Control
Source: Biomolecules. 2026 Oct 2;16(10):1433. doi: 10.3390/biom16101433 (PMC13702173; doi:10.3390/biom16101433)
Supplement: Supplementary file 1 [file biomolecules-16-01433-s001.zip › biomolecules-4537538-supplementary.pdf]

Supplementary Tables

Longitudinal Circulating Nerve Growth Factor in Pancreatic Ductal Adenocarcinoma: An Exploratory Study of Postoperative Recurrence and First-Line Disease Control

Table S1. Phase-aligned circulating NGF association screen.

Timepoint	NGF measure	Outcome/variable	Test	N	Effect size	p	q
Postoperative	NGF-dopo chirurgia	Recurrence	Mann-Whitney	19	0.467	0.033	0.542
Postoperative	NGF-dopo chirurgia	Pain	Mann-Whitney	20	1.000	0.052	0.542
First-line start	NGF-inizio prima linea	Disease status	Permutation Kruskal-Wallis	40		0.079	0.542
First-line progression	NGF-PD 1 linea	CA19-9 at progression	Permutation Spearman	15	0.455	0.095	0.542
First-line start	NGF-inizio prima linea	PFS 1L	Permutation Spearman	30	-0.309	0.098	0.542
Recurrence/end adjuvant	NGF-alla recidiva	Pain	Mann-Whitney	10	1.000	0.101	0.542
First-line progression	NGF-PD 1 linea	PFS 1L	Permutation Spearman	18	-0.391	0.109	0.542
Recurrence/end adjuvant	NGF-alla recidiva	DFS	Permutation Spearman	9	-0.572	0.111	0.542
First-line start	NGF-inizio prima linea	CEA at first-line start	Permutation Spearman	31	-0.261	0.151	0.597
First-line progression	NGF-PD 1 linea	CEA at progression	Permutation Spearman	15	0.392	0.153	0.597
First-line progression	NGF-PD 1 linea	PD-L1 at progression, plasma	Permutation Spearman	14	-0.355	0.214	0.679
Postoperative	NGF-dopo chirurgia	CA19-9 after surgery	Permutation Spearman	20	0.293	0.222	0.679
Recurrence/end adjuvant	NGF-alla recidiva	CEA at recurrence	Permutation Spearman	8	-0.475	0.226	0.679
Postoperative	NGF-dopo chirurgia	N stage	Permutation Kruskal-Wallis	20		0.251	0.700
Postoperative	NGF-dopo chirurgia	Pathological PNI	Mann-Whitney	20	0.375	0.284	0.729
Postoperative	NGF-dopo chirurgia	DFS	Permutation Spearman	10	-0.344	0.316	0.729
Postoperative	NGF-dopo chirurgia	PD-L1 after surgery, plasma	Permutation Spearman	10	0.348	0.318	0.729
First-line progression	NGF-PD 1 linea	TGF-beta at progression, serum	Permutation Spearman	9	0.365	0.359	0.779
Postoperative	NGF-dopo chirurgia	TGF-beta after surgery, plasma	Permutation Spearman	10	0.307	0.386	0.791
First-line start	NGF-inizio prima linea	TGF-beta at first-line start, plasma	Permutation Spearman	18	0.257	0.452	0.863
First-line start	NGF-inizio prima linea	Pain	Mann-Whitney	40	-0.103	0.465	0.863

Postoperative	NGF-dopo chirurgia	CEA after surgery	Permutation Spearman	20	0.166	0.513	0.863
Postoperative	NGF-dopo chirurgia	T stage	Permutation Kruskal-Wallis	20		0.530	0.863
Postoperative	NGF-dopo chirurgia	OS	Permutation Spearman	20	0.143	0.552	0.863
First-line progression	NGF-PD 1 linea	PD-L1 at progression, serum	Permutation Spearman	9	-0.251	0.553	0.863
Recurrence/end adjuvant	NGF-alla recidiva	OS	Permutation Spearman	10	-0.200	0.583	0.874
First-line start	NGF-inizio prima linea	PD-L1 at first-line start, plasma	Permutation Spearman	18	0.187	0.613	0.884
First-line progression	NGF-PD 1 linea	OS	Permutation Spearman	18	-0.124	0.634	0.884
First-line start	NGF-inizio prima linea	PD-L1 at first-line start, serum	Permutation Spearman	12	0.219	0.673	0.905
First-line start	NGF-inizio prima linea	OS	Permutation Spearman	40	0.042	0.796	0.931
First-line start	NGF-inizio prima linea	CA19-9 at first-line start	Permutation Spearman	31	0.041	0.819	0.931
Postoperative	NGF-dopo chirurgia	TGF-beta after surgery, serum	Permutation Spearman	4	0.211	0.832	0.931
Postoperative	NGF-dopo chirurgia	PD-L1 after surgery, serum	Permutation Spearman	4	0.211	0.833	0.931
Recurrence/end adjuvant	NGF-alla recidiva	PD-L1 at recurrence/end adjuvant, plasma	Permutation Spearman	5	-0.154	0.837	0.931
First-line progression	NGF-PD 1 linea	Pain	Mann-Whitney	18	0.050	0.843	0.931
Recurrence/end adjuvant	NGF-alla recidiva	CA19-9 at recurrence	Permutation Spearman	8	0.065	0.888	0.931
Recurrence/end adjuvant	NGF-alla recidiva	TGF-beta at recurrence/end adjuvant, plasma	Permutation Spearman	5	0.103	0.900	0.931
First-line progression	NGF-PD 1 linea	TGF-beta at progression, plasma	Permutation Spearman	14	0.036	0.907	0.931
First-line start	NGF-inizio prima linea	TGF-beta at first-line start, serum	Permutation Spearman	12	0.044	1.000	1.000
Recurrence/end adjuvant	NGF-alla recidiva	TGF-beta at recurrence/end adjuvant, serum	Permutation Spearman	2			
Recurrence/end adjuvant	NGF-alla recidiva	PD-L1 at recurrence/end adjuvant, serum	Permutation Spearman	2			

Note: Effect size is Spearman rho for continuous variables and rank-biserial r for Mann-Whitney tests; no single effect-size estimate is shown for Kruskal-Wallis tests. q values are Benjamini-Hochberg false-discovery-rate adjusted.

Table S2. Sensitivity analyses comparing postoperative and active-disease circulating NGF measurements.

Comparison	Postoperative N	Postoperative NGF median (IQR)	Comparator N	Comparator NGF median (IQR)	Effect size	p	q
Postoperative vs all active-disease measurements	20	0.0 (0.0-31.9)	70	0.0 (0.0-12.9)	-0.014	0.920	0.920
Postoperative vs recurrence	20	0.0 (0.0-31.9)	10	81.6 (0.0-283.5)	0.240	0.243	0.920
Postoperative vs first-line start	20	0.0 (0.0-31.9)	40	0.0 (0.0-0.0)	-0.050	0.715	0.920
Postoperative vs first-line progression	20	0.0 (0.0-31.9)	18	0.0 (0.0-5.0)	-0.044	0.784	0.920

Note: Effect size is rank-biserial r comparing comparator group with postoperative measurements. q values are Benjamini-Hochberg false-discovery-rate adjusted.
